# Silence sexual and reproductive health discussions and we fuel the rise of HIV/AIDS in sub-Saharan Africa

**DOI:** 10.1186/s12978-017-0395-1

**Published:** 2017-10-17

**Authors:** Janet M. Wojcicki

**Affiliations:** Department of Pediatrics, University of California, 550 16th Street, San Francisco, CA 94134-0136 USA

## Abstract

In the mid 1990s, the HIV epidemic was initially impacting South Africa. Fear, stigma and denial surrounding sexual practices undermined treatment access and prevention initiatives. Significant strides have been made in reducing the HIV epidemic in South Africa and other areas in sub-Saharan Africa through effective programming and funding of prevention programs. Reinstatement of the Mexico City Policy threatens to negatively impact gains made in the HIV/AIDS community. Recognition that communication is essential to effective reproductive health and HIV/AIDS programming needs to be recognized by politicians enacting the Mexico City Policy and the possibility of viewing a rise in HIV/AIDS incidence in sub-Saharan Africa.

## Background

### South Africa and HIV/AIDS

I worked as an anthropologist interviewing commercial and informal sex-workers in Johannesburg and Soweto, South Africa in the mid to late 1990s. During this time, as South Africa was emerging from decades of apartheid legislation, and into the brutal reality of the HIV/AIDS epidemic, stigma surrounding diagnosis and testing were fueling the spread of HIV. These were brutal times - the epidemic of HIV/AIDS exploded from less than 1% of the population infected in 1990 to more than 20% in 2000. The commercial and informal sex workers who I interviewed were often not seeking HIV testing, counseling or treatment because of their fears of derogatory comments and judgments from health workers [1, 2].

Since the mid 1990s, South Africa, as the country with the largest number of individuals living with HIV and AIDS (6.1 in 2012 and 7 million in 2015) [3, 4], has made significant strides in the fight against HIV/AIDS. Indeed, some of this can be explained by the emergence of access to antiretroviral therapies (ARTs) but perhaps, more importantly, there has been a widespread effort to de-stigmatize sexual behaviors and marginalized population groups and increase the uptake of voluntary testing and counseling [5] although this work is by no means complete [6]. The National Institute of Health (NIH), US federal and other governmental and private foundation have helped in this process by funding interventions to reduce stigma and organizations that counsel on reproductive and sexual health as a means to encourage testing and treatment of HIV/AIDS.

Meanwhile, however, these problems persist and continue and HIV-related stigma and isolation impacts those infected [7] as well as prevents access to services for those who may not know infection status [8]. The WHO cites stigma and fear as the main reasons that individuals are reluctant to get tested, disclose their status and take ARTs [9]. Specifically, one of the most significant barriers to ART adherence is on-going negative experience and perceived stigma from healthcare providers. Reluctance to get tested and initiate treatment results in increased risky behaviors and spread of disease to others [9].

### South Africa and reproductive health counseling

Unlike other sub-Saharan African countries, South Africa is relatively unique in the region and in sub-Saharan Africa and elsewhere in being one of a few that provides legal termination of pregnancy via the Choice of Termination of Pregnancy Act, Act 92 of 1996 (the others include Mozambique, Cape Verde and Zambia). One out of every three women aged 15–24 experiences unintended pregnancy before the age of 20 in South Africa. In Southern Africa in general, approximately 59% of pregnancies are unintended [10]. With legalized abortion, there can be open dialogue about different options for women. However, South Africa still has considerable distance to go. Maternal morbidity is high as approximately 50% of abortions are provided outside of clinic or hospital settings because of lack of information and stigma from the medical community and shame. Throughout sub-Saharan Africa and elsewhere there is an on-going struggle of reaching out to women, particularly those from marginalized population groups because of fear, mistrust and high levels of stigma associated with termination of pregnancy [11].

The United States has reinstated the Mexico City Policy or “Global Gag Rule” which prevents access to US federal funds for any organization that offers or advises on family planning and reproductive health options if it includes information on abortion, even if these funds are not used for abortion-related services. The United States funds approximately $607.5 million for family planning assistance every year and the new restrictions would negatively impact a sum of $8.5 billion dollars, which goes to global health in general (over 50% of this for HIV/AIDS services). In many sub-Saharan African communities, uptake of contraceptive services is low as are interactions with the healthcare community, in part due to on-going stigma. Studies have shown that those organizations, which decide to forego the US funding will havefewer resources for family planning and contraceptive services in general including general HIV prevention [10].

Discussion and open communication is the key to bridging the gap between marginalized communities and access to healthcare including voluntary HIV testing and counseling, access to ARTs and other medications. There is still much work to be done to reduce the stigma and fear that communities face in their interactions with healthcare workers. The answer is not to shut off communications in one area. Pregnancy and unintended pregnancy are part of a woman’s reproductive health and by denying counseling and discussion in this and associated areas, we will push away women and communities who need our services.

## Conclusions

### South Africa and the future

South Africa represents some of the hope for the African continent as the laws here and the constitution protect the rights of women even if they are not universally enforced as domestic violence, rape and other acts against the rights of women are at high levels. However, even in South Africa, there are on-going challenges of providing quality reproductive, HIV and sexually transmitted infection (STI) health care to women due to stigma [6]. Open communication and trust is the answer to bridge these divides and we need to go forward providing more comprehensive services and information, not less.

## Abbreviations

HIV/AIDS: Human Immunodeficiency Virus/Acquired Immunodeficiency Virus
